# Lyso-glycosphingolipids: presence and consequences

**DOI:** 10.1042/EBC20190090

**Published:** 2020-08-18

**Authors:** Marco van Eijk, Maria J. Ferraz, Rolf G. Boot, Johannes M.F.G. Aerts

**Affiliations:** Leiden Institute of Chemistry, Leiden University, Einsteinweg 55, 2300 RA, Leiden, The Netherlands

**Keywords:** acid ceramidase, glycosphingolipid, immune response, lysosomal storage disease

## Abstract

Lyso-glycosphingolipids are generated in excess in glycosphingolipid storage disorders. In the course of these pathologies glycosylated sphingolipid species accumulate within lysosomes due to flaws in the respective lipid degrading machinery. Deacylation of accumulating glycosphingolipids drives the formation of lyso-glycosphingolipids. In lysosomal storage diseases such as Gaucher Disease, Fabry Disease, Krabbe disease, GM1 -and GM2 gangliosidosis, Niemann Pick type C and Metachromatic leukodystrophy massive intra-lysosomal glycosphingolipid accumulation occurs. The lysosomal enzyme acid ceramidase generates the deacylated lyso-glycosphingolipid species. This review discusses how the various lyso-glycosphingolipids are synthesized, how they may contribute to abnormal immunity in glycosphingolipid storing lysosomal diseases and what therapeutic opportunities exist.

## Introduction

In 1884 the German chemist and clinician J.L.W. Thudichum described a new class of lipids, now known as glycosphingolipids (GSLs), when studying the composition of brain [1]. This enigmatic class consists of a sphingoid base attached to an acyl chain and a carbohydrate moiety (for detailed review see Merrill) [2]. GSLs are involved in a plethora of physiological processes and pathologies [3–5]. GSLs are essential components of the outer leaflet of cell membranes. As constituents of glycosphingolipid enriched microdomains GSLs can contribute to signaling processes. The ganglioside GM3, a complex GSL, has for instance been shown to modulate epidermal growth factor (EGF)-R -and insulin-R signaling [6,7]. GSLs are also involved in pathogen recognition and can serve as entry point of virus (GM1 acts as receptor for simian virus 40 (SV40) and other Polyomavirus) and bacteria (GM1 acts as receptor for various bacteria) or can serve as toxin binding site (GB3 binds to shiga toxin and *Escherichia coli* derived verotoxin B subunit) (recently reviewed by Aerts) [3,5]. Complex GSLs have also been connected to CD4^+^ and CD8^+^ lymphocyte function. Mice lacking GM3 synthase show severely diminished CD4^+^ T-cell activation, without disturbance of CD8^+^ T-cell activation. Vice versa GM2/GD2 synthase deficient mice show absent CD8^+^ T-cell activation, with normal CD4^+^ T-cell activation. Interestingly, GM3 synthase lacking mice are not developing ovalbumin (OVA) induced asthma [8]. A homozygous loss of function in the GM3 synthase gene causes an epilepsy syndrome in men [9]. GSLs are also part of the ABO blood group antigens that are critical mediators in transfusion medication [10]. As key constituents of the myelin sheet galactosylceramide and sulfatides have been reported to contribute to its stability and continuity [11].

**Figure 1:**
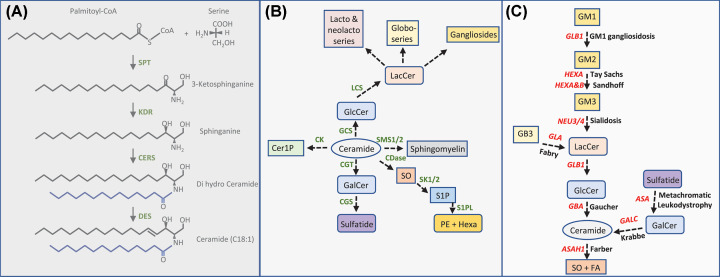
Metabolism of glycosphingolipids (**A**) Schematic representation of the *de novo* synthesis of sphingolipids. **SPT**: serine palmitoyltransferase. **KDR**: ketoreductase. **CERS**: ceramide synthase. **DES**: desaturase. The acylchain is depicted in blue and can range from C14-C26 depending on the CERS enzyme involved in acyl chain condensation (six CERS members exist). Depending on the acyl-CoA moiety used by SPT, i.e. myristoyl-CoA, palmitoyl-CoA or stearyl-CoA different sphingoid bases can be produced (C16:1, C18:1 or C20:1). (**B**) Generation of ceramide derived species including glycosphingolipids. **CK**: ceramide kinase. **GCS**: glucosylceramide synthase. **SMS**: sphingomyelin synthase. **CGT**: UDP-galactose ceramide galactosyl transferase. **CGS**: cerebroside sulfotransferase. **CDase**: ceramidase. **SK**: sphingosine kinase. **S1PL**: S1P lyase. **LCS**: lactosylceramide synthase. **Cer1P**: ceramide-1-phosphate. **GlcCer**: glucosylceramide. **LacCer**: lactosylceramide. **GalCer**: galactosylceramide. **SO**: sphingosine. **S1P**: sphingosine-1-phosphate. **PE**: phosphatidylethanolamine. **Hexa**: hexadecanal. (**C**) Lysosomal degradation of GSL with responsible gene products and diseases connected to defects. *GLB1* (β-galactosidase), *HEXA* (β-hexosaminidase A), *HEXB* (β-hexosaminidase B), *NEU3/4* (neuraminidase 3/4), *GLA* (α-galactosidase A), *GBA* (β-glucocerebrosidase), *ASA* (Arylsulfatase A), *GALC* (β-galactosylceramidase), *ASAH1* (acid ceramidase).

To synthesize GSLs, first ceramide has to be made [2,12]. Formation of this key sphingolipid starts at the endoplasmic reticulum (ER) with the condensation of L-serine to palmitoyl-CoA, which depends on the activity of serine palmityoltransferase (SPT), yielding 3-ketosphinganine (Figure 1A,B). Subsequent reduction by ketosphinganine reductase (KDR) gives rise to sphinganine and further addition of an acyl chain by one of the ceramide synthases (CERS) produces dihydroceramide. Depending on the CERS enzyme (6 members exist) fatty acyl chains with various lengths can be incorporated [13,14]. By the action of dihydroceramide desaturase (DES) ceramide is formed, consisting of a C18:1 sphingoid base and a fatty acyl chain with a length ranging from C14-C26. Alternatives to this route can be introduced when other fatty acyl CoA variants are used by SPT such as myristoyl-CoA (yielding ceramide with a C16:1 sphingoid base), or stearyl-CoA (yielding ceramide with a C20:1 sphingoid base) [12]. Instead of serine, also L-alanine or glycine can be incorporated by SPT and this will yield uncommon 1-deoxysphingolipids. Due to the absence of the hydroxyl group at the C1 position these species cannot be metabolized further. It is hypothesized that the unfavorable use of L-alanine over L-serine is caused by certain SPT variants and these are associated with hereditary sensory and autonomic neuropathy type I (HSAN-1) [15]. A clear physiological role has not yet been established for 1-deoxysphingolipids.

GSLs arise from ceramide following the addition of a sugar moiety/various sugar moieties. The most fundamental GLSs are glucosylceramide (GlcCer) and galactosylceramide (GalCer), which are synthesized upon condensation of UDP-glucose, or UDP-galactose to ceramide being catalyzed by glucosyl/galactosyl ceramide synthase (Figure 1B). Further carbohydrate addition allows for the generation of sulfatide, ganglioside, globoside, isogloboside, lacto and neolacto species [2].

Alternative fates of ceramide can be summarized as follows. Ceramide can be converted into sphingomyelin (SM) by the action of SM synthase 1/2. Deacylation of ceramide by ceramidase (CDase) yields free fatty acid and sphingosine, of which sphingosine can be further phosphorylated by sphingosine kinase 1/2 forming sphingosine-1-phosphate (S1P). S1P can be degraded by S1P lyase. Direct phosphorylation of ceramide by ceramide kinase (CK) drives ceramide-1-phosphate (C1P) formation [12,16].

**Figure 2:**
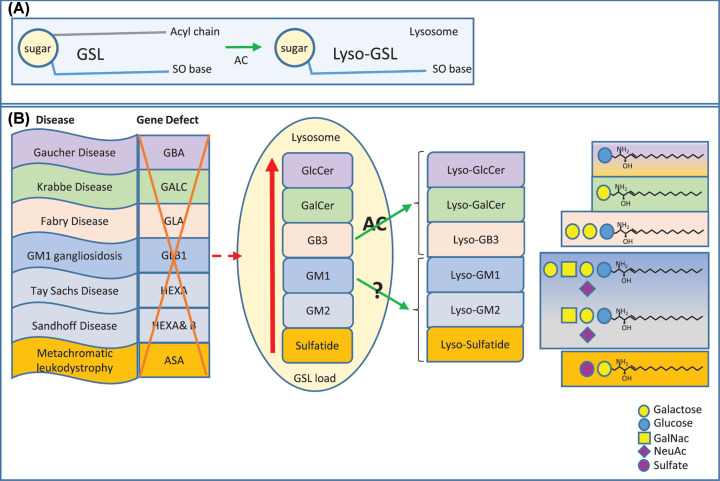
Generation of lyso-glycosphingolipids Schematic representation of (**A**). The generation of lyso-GSL by the action of acid ceramidase (**AC**). (**B**) From left to right. The lysosomal storage disorders, the gene defect, accumulating lysosomal GSL substrates and the deacylated lyso-GSL species. **GBA**, acid β-glucosidase. GALC, β-galactosylceramidase. **GLA**, α-galactosidase A. **GLB1**, β-galactosidase. **HEX**, β-hexosaminidase. **ASA**, Arylsulfatase A. **GlcCer**, glucosylceramide. **GalCer**, galactosylceramide. **GB3**, globotriaosylceramide. **GM1**, ganglioside GM1. **GM2**, ganglioside GM2. **SO**, sphingoid.

As a result of endocytosis or autophagy of cellular membranes, or for instance phagocytosis of bacteria, GSL substrates enter the endo-lysosomal system and their degradation occurs in lysosomes in a step-wise fashion (Figure 1C). Specific glycosidases trim the GSLs with the help of so-called activator proteins (GM2 activator protein or saposins A-D) [17]. The final action of acid ceramidase gives rise to the formation of sphingosine. Importantly, sphingosine can re-enter a recycling pathway, also referred to as salvage pathway. Re-acylation results in ceramide formation [18,19]. Failure to degrade GSLs gives rise to lysosomal GSL storage disorders such as Gaucher Disease, Fabry Disease and Krabbe Disease (details will be discussed later). Intra-lysosomal accumulation of GSLs provides substrate for acid ceramidase that as a consequence of its deacylation activity gives rise to the formation of lyso-glycosphingolipid (lyso-GSL) species (Figure 2A).

In this review, it is addressed how lyso-GSL species accumulate during lysosomal storage disorders, how they contribute to immunity and therapeutic options are discussed.

## Glycosphingolipid storage disorders

Lysosomal storage disorders (LSDs) are caused by defects in the lysosomal catabolic machinery, either caused by mutations in hydrolases, defective transport molecules or dysfunctional accessory proteins (saposins for instance) [20,21]. GSL storage disorders discussed in this review are: Gaucher Disease (caused by deficiency in lysosomal acid β-glucosidase/glucocerebrosidase (EC3.2.1.45), *GBA* gene). Fabry Disease (caused by deficiency in lysosomal α-galactosidase A (EC3.2.1.22), *GLA* gene), Krabbe disease/globoid cell leukodystrophy (caused by deficiency in lysosomal β-galactosylceramidase (EC3.2.1.46), *GALC* gene), GM1 gangliosidosis (caused by deficiency in lysosomal β-galactosidase (EC3.2.1.23), *GLB1* gene) and the GM2 gangliosidosis Tay Sachs Disease (caused by deficiency in lysosomal β-hexosaminidase (EC3.2.1.52) A, *HEXA* gene) and Sandhoff Disease (caused by deficiency in lysosomal β-hexosominidase A & B, *HEXA* & *HEXB* gene) and Metachromatic leukodystrophy (caused by deficiency in lysosomal Arylsulfatase A (EC3.1.6.8) *ASA* gene). Niemann Pick type C is caused by deficient intracellular cholesterol transporter proteins (NPC1/2), which gives rise to secondary GSL accumulation most likely caused by a general impairment of lysosomal hydrolase action will also be reviewed [20–22] (see Figure 2B).

## Lyso-glycosphingolipids

Several lyso-GSL species, also referred to as glycosphingoid bases, have been reported in LSD wherein specific GSLs accumulate in lysosomes. The LSD and corresponding accumulating lyso-GSL, including their alternative names, are depicted in Table 1 and Figure 2. The storing lysosomal GSL species become susceptible to acid ceramidase (also named N-acylsphingosine deacylase (EC 3.5.1.23), *ASAH1* gene) action allowing the formation of lyso-GSL (see Figure 2) as is evidenced by both *in vivo* and *in vitro* studies. Formation of glucosylsphingosine (GlcSph) and Lyso-globotriaosylsphingosine (lyso-GB3) depends on acid ceramidase activity as can be concluded from studies using acid ceramidase deficient Farber Disease fibroblasts, studies with the acid ceramidase inhibitor carmofur (an organohalogen compound that also can trigger the generation of 5-FU, a pyrimidine analogue) and studies using isotope labeled GSL species [23,24]. Furthermore, formation of galactosylsphingosine (GalSph)/psychosine in Krabbe Disease requires the activity of acid ceramidase as is evidenced recently in studies using twitcher mice (Krabbe disease model) in which acid ceramidase activity was ablated genetically. This was achieved by crossing twitcher mice with acid ceramidase deficient mice (Farber Disease mice), resulting in the elimination of psychosine accumulation and Krabbe Disease. In addition, carmofur extended life span of twitcher mice and in Krabbe disease patient fibroblasts psychosine levels were lowered by using the same inhibitor [25]. Taken together, it is highly likely that any accumulating GSL substrate becomes deacylated by acid ceramidase, to its respective deacylated form, the lyso variant. Of note, this has been proven for GlcSph, GalSph and lyso-GB3, but for other GSL substrates such as sulfatide and gangliosides this remains to be experimentally determined.

**Table 1:** Glycosphingolipid storage disorders and accumulating lyso-GSL species

Lysosomal storage disorder	Accumulating lyso-GSL and aliases
Gaucher disease	Glucosylsphingosine (GlcSph), lyso-glucosylceramide (LGL1), Globotriaosylsphingosine (lyso-GB3, lyso-CTH)
Fabry disease	Globotriaosylsphingosine (lyso-GB3, lyso-CTH)
Krabbe disease/globoid cell leukodystrophy/ galactosylceramide lipidosis	Galactosylsphingosine (GalSph, lyso-GalCer, psychosine)
Niemann Pick type C	Glucosylsphingosine (GlcSph, lyso-GlcCer, lyso-GL1) (Phosphorylcholinesphingosine (lyso-S(P)M))
GM1 gangliosidosis GM2 gangliosidosis (Tay Sachs, Sandhoff Disease)	Lyso-monosialoganglioside GM1 (lyso-GM1), lyso-GA1 Lyso-monosialoganglioside GM2 (lyso-GM2), lyso-GA2
Metachromatic leukodystrophy (MLD)	Sulfogalactosylsphingosine (lyso-sulfatide)

The fate of a lyso-GSL also depends on the existence of additional non-lysosomal catabolic activity. In the case of GlcSph the altered chemical properties allow for lysosomal escape and as a consequence GlcSph can become substrate to the non-lysosomal glucosylceramidase GBA2. Supposedly, this enzyme can remove the glucose group thereby generating a local pool of sphingosine in the plasma membrane that can be further metabolized by for instance sphingosine kinase 1 [12,26]. Interestingly, both lysosomal GBA and non-lysosomal GBA2 not only act as hydrolases, but also can act as transglycosylases. This allows for removal of glucose from GlcSph/GlcCer, that subsequently can be transferred to an available acceptor. An example of this reaction is the formation of glucosylated cholesterol (GlcChol) [27]. In theory, lyso-GSLs might be re-acylated in the cytosol, but direct evidence for such reaction is still lacking. The various lyso-GSLs arising during LSD and their impact on inflammation will be discussed below.

## Lyso-GSLs in immunity

**Figure 3:**
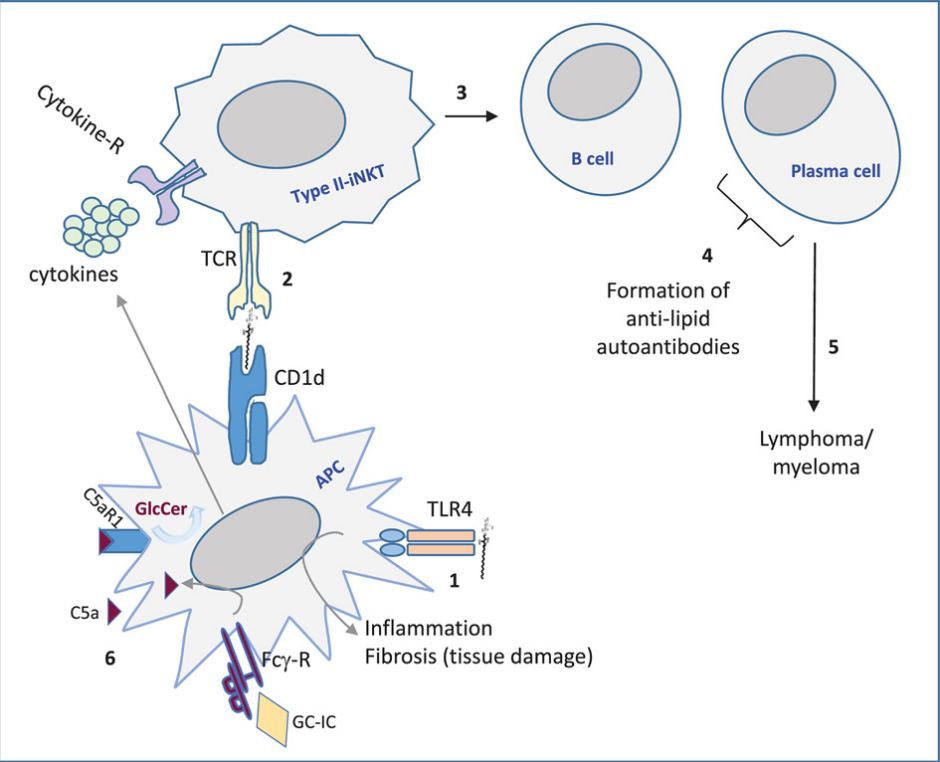
Simplified model of immune activation by (lyso)-GSL in LSD **1.** Direct recognition of lyso-GSL by toll-like receptor 4 (TLR4). **2.** CD1d-restricted lyso-GSL presentation and activation of type II iNKT cells. **3.** iNKT cell-mediated activation of B cells and plasma cells. **4.** Production of anti-(lyso)-GSL autoantibodies by plasma cells. **5.** Continuous antibody production may result in genetic instability driving cancer cell formation. **6.** Glucosylceramide (GC)-immune complexes (IC) interact with Fcγ-R inducing C5a (part of complement system), which by binding to C5aR1 triggers induction of more GlcCer (and connected GlcSph). APC, antigen presenting cell.

The least complex sphingoid base is sphingosine (SO), which arises upon deacylation of ceramide by ceramidase action. At least five ceramidases exist with different cellular locations and pH optimums [12]. As mentioned earlier, this review is centered around the lyso-GSLs that increase during LSD and how they impact immunity. A consequence of any (lyso)-GSL accumulating disease could be that lipid-mediated immunity becomes different. Not only GSL load changes, also novel lipid species, for instance the lyso-GSL, or unexpected glucosylated variants such as GlcChol, arise and all may induce immune activation. A possible explanation for the latter activation is that these unique lipids were absent during selection of the immune repertoire, or exceed a threshold and become recognized. Generalizing it can be stated that storage lipids, or derived metabolites, can be presented in CD1d molecules by antigen presenting cells (APC) to Natural Killer (NK) T cells. Invariant/type I NK T cells, or type II NK T cells can recognize the presented CD1d-lipid complex in their T-cell receptor (TCR). Type I NK T cells recognize the potent lipid antigen α-galactosylceramide (α-GalCer), whereas type II NKT do not respond to α-linked glycolipids [28,29]. Subsequently, the lipid antigen activated NKT cells may instruct other immune cells, such as B cells, driving formation of anti-lipid autoantibodies. Furthermore, recognition of abnormal levels of (lyso)-GSL via toll-like receptors (TLR) may occur. For an overall model see Figure 3. Lastly, complex GSLs can impact lymphocyte function. For instance the earlier discussed activation of CD4 T lymphocytes requires GM3 synthase activity and CD8 T lymphocyte activation requires GM2/GD2 synthase activity [8]. In the next sections, it will be described how different disease related lyso-GSL species influence immunity.

## Galactosylsphingosine/psychosine (Krabbe disease)

In the early 70s, it was discovered that Krabbe disease/globoid cell leukodystrophy was caused by deficiency in the enzyme β-galactosidase (GALC) that normally degrades galactosylceramide [30]. The “psychosine hypothesis" followed soon and herein it was formulated that psychosine accumulation caused the clinical symptoms connected to Krabbe disease [31,32]. This was further extended on by the detailed structural analysis of isolated psychosine [33]. Psychosine causes detrimental progressive neurological damage, for instance reflected by severe demyelination [31]. Recently, it became clear that lysosomal acid ceramidase deacylates GalCer to generate psychosine. The seminal work in 1987 by Hannun revealed that a multitude of lyso-GSL, including psychosine, inhibited protein kinase C activity due to the presence of a basic amine and the amphipathic nature of these lipids [34]. Furthermore, it has been shown that psychosine perturbs membrane organization by acting on lipid rafts and inhibits fast axonal transport [35,36]. As a consequence, myelin component organization, synapse and axonal function and immunity all may be perturbed. Krabbe disease patients and primate, mouse and fish disease models display increased central nerve nervous system inflammation characterized by myelin debris clearing macrophages (unique globoid cells) and an increase in B and T cells and inflammatory cytokines and chemokines [37]. How exactly psychosine contributes to inflammation is not fully resolved. Besides induction of apoptosis, direct immunomodulatory effects have also been reported, which can be summarized as follows. *In vitro* studies using peripheral blood mononuclear cells (PBMCs) of Krabbe disease patients revealed that stimulation with lipopolysaccharide (LPS) did not induce significantly different tumor necrosis factor (TNF)α levels compared with controls, but an additive effect of low psychosine on TNFα was observed in Krabbe patients [38,39]. At higher psychosine concentration the difference was not significant, presumably due to toxicity [39]. Furthermore, it has been shown *in vitro* that psychosine can trigger multi-nucleated globoid like cell formation when added to human myelomonocyte U937 cells [40]. In astrocytes induction of inflammation by psychosine occurred through inactivation of AMP-activated protein kinase (AMPK). Interestingly, activation of AMPK using 5-aminoimidazole-4-carboxamide-1-β-D-ribofuranoside (AICAR) lowered nitric oxide (NO) production and pro-inflammatory factors iNOS and Cox-2 [41].

## Glucosylsphingosine (Gaucher Disease and NPC)

Gaucher Disease (GD) is caused by glucocerebrosidase deficiency and owes its name to the French clinician Philippe Gaucher who first reported on the disease at the end of the 19th century [42]. Almost a century later the biochemistry (GlcCer accumulation) and genetics (mutations in the *GBA* gene) were solved [43,44]. GD occurs in three forms, namely the most common type 1 (non-neuronopathic), a type 2 (acute neuronopathic) and a type 3 (sub-acute neuronopathic) variant. Major symptoms encountered in the non-neuronopathic form of GD can be summarized as follows: hepatosplenomegaly, low blood platelet count, blood clotting abnormalities, anemia and bone disease [4]. An important immune component contributing to GD pathology is steered by macrophages. Being professional phagocytes implies that a lot of lipid substrates enter the macrophages for degradation and thus flaws in the catabolic machinery easily will translate into a phenotype. Macrophages are lipid-laden due to accumulation of GlcCer and are referred to as Gaucher cells [4]. Not surprisingly, Gaucher cell derived circulating proteins can aid in disease determination and therapy monitoring, exemplified for instance by chitotriosidase (FDA approved disease marker), the chemokine (C-C motif) ligand 18 (CCL-18) and glycoprotein nonmetastatic melanoma protein B (GPNMB) [45–47]. The lysosomal accumulation of GlcCer induces acid ceramidase assisted GlcSph formation [24,48]. The accumulation of lysolipids in LSDs in absolute amounts is very small compared with that of the parent sphingolipids, indicating that only a fraction of the latter is converted by acid ceramidase [24]. The increased polarity of lyso-GSL allows their transport in blood and consequently activity distant from the site where it is produced. Supposedly, Gaucher cell-derived GlcSph can act both in an autocrine and paracrine fashion. Already in the early 70’s and 80’s GlcSph was found to be elevated in cells and tissues of GD patients [49,50]. GlcSph has been found to effect *in vitro* osteoblast function and to induce neuronal cell line dysfunction [51,52]. Furthermore, it has been speculated that GlcSph contributes to neuropathology [53,54]. Possibly, GlcSph induces neuronal death by acting on Ca^2+^ mobilization as was demonstrated in rat brain microsomes [55]. Carriers of mutant GBA allele are at considerably increased risk to develop Parkinson disease [56]. GlcSph has been shown to stimulate pathological α-synuclein aggregation [57,58]. GlcSph is markedly elevated in plasma of GD patients, a hallmark exploited for diagnostic purposes. Plasma GlcSph levels correlate well with those of established protein biomarkers of Gaucher cells such as chitotriosidase and CCL18, suggesting that GlcSph is predominantly macrophage derived [48]. The presence of excessive GlcSph was also recapitulated in various GD animal models, including mouse, drosophila melanogaster, sheep and zebrafish [59–62]. A direct effect of GlcSph has been studied in C57BL/6JRj mice. Subcutaneous GlcSph infusion (10 mg/kg/day) for up to 12 weeks resulted in plasma concentrations of 700–900 ng/ml and strong elevation of the lipid in all peripheral tissues, with only a very modest increase in brain. Hemoglobin levels and hematocrit levels (except the 12-week point) significantly dropped in response to the GlcSph challenge. Spleens enlarged, which coincided with increased macrophage content. In plasma pro-inflammatory cytokines TNFα, interleukin (IL)-1β and IL-23 were slightly elevated after 12 weeks [63]. A remark here is that the animals were exposed to very high levels of GlcSph. Recently, a key role for compliment activation was observed in the Gba^19V/-^ GD mouse model. Increased C5a levels were detected in sera, produced by dendritic cells (DCs) and macrophages. C5aR1, induced on DCs and macrophages, turned out to be the key sensor of C5a. The C5a-C5aR1 loop triggers induction of co-stimulatory molecules on DCs (CD40, CD80 and CD86) and CD40L and CD69 on CD4 T cells all contributing to a pro-inflammatory environment (IFNγ, TNFα, IL-1β, IL-6 and IL-17A/F). Importantly, the C5a-C5aR1 axis also controls GlcCer production, both at steady state level and during pathology. GlcCer specific auto-antibody immune complexes (IC) boost the C5a axis upon Fcγ-R triggering on macrophages. In GD patient sera C5a levels were increased, GlcCer specific auto-antibodies were present and *in vitro* GlcCer IC induced C5a, CCL18 and several pro inflammatory cytokines in U937 cells [64]. Not only GlcCer serves to be a lipid auto-antigen, but also GlcSph. Excessive, immunogenic GlcSph has been postulated to underly the gammopathies commonly encountered in Gaucher patients that may develop into multiple myeloma, a relatively common leukemia in patients with GD [65]. Immunoglobulin reactivity against GlcSph was detected in a GD mouse model and in patients with monoclonal -and polyclonal gammopathy. Injection of GlcSph into young GBA^-/-^ mice triggered an increase in splenic germinal center B cells and bone marrow plasma cells and concomitantly results in more anti-GlcSph antibodies [65]. Previously it was already found that a novel subset of type II NKT cells showed reactivity to the GD lipids β-GlcCer 22:0 and GlcSph. These type II NK T cells also expressed markers of T-follicular helper cells and are referred to as type II NKT-TFH. The CD1d restricted presentation of Gaucher Disease lipids allows for NKT activation, followed by germinal center B-cell activation and anti-lipid antibody production [66]. A different way of explaining the mechanisms underlying the common gammopathy and high incidence of multiple myeoloma has also been published. Preuss et al. presented data suggesting that saposin C, the activator of glucocerebrosidase in lysosomes, acts as autoantigen, driving B-cell activation [67]. A first attempt to reproduce this finding was not successful, but this alternative mechanism requires further investigation [68]. Another considered toxic effect of GlcSph is interference with endothelial cytokinesis that might explain the reduced cerebral microvascular density neuronopathic Gaucher mice [69]. Earlier *in vitro* studies have suggested that excessive GlcSph might cause lysis of red blood cells, impair cell fission during cytokinesis, interfere with growth, and promote inflammation via activation of phospholipase A2 [70]. All these findings are consistent with signs and symptoms in patients with GD such as occurrence of hemolysis, multinucleated macrophages, growth retardation, and chronic low-grade inflammation [70]. Taken together, GlcCer and GlcSph contribute to auto immunity observed in GD. Interestingly, in Niemann Pick type C (NPC) not only GSLs, but also GlcSph is increased [22,26,71]. Although the immune system is altered in NPC (reviewed by Platt), no clear role for GlcSph has been demonstrated yet [72]. iNKT cell are virtually absent in mouse models of NPC, whereas numbers are normal in human NPC patients [73,74]. A possible explanation for the differences observed between GD and NPC with respect to the response to the elevated levels of GlcSph may be the absolute numbers in plasma, which are 50- to 100-fold lower in NPC. The generalized lysosomal dysfunction occurring in NPC may also impact acid ceramidase activity and thereby the capacity to generate GlcSph.

## Globotriaosylsphingosine/lyso-GB3 (Fabry disease)

The X-linked LSD Fabry disease is caused by flaws in the activity of α-galactosidase A and a high level of the storage product GB3 is found in endothelial cells [70]. GB3 accumulation is associated with renal failure, cardiac problems and central nervous system pathology [70]. An important study demonstrated that lyso-GB3 was elevated in symptomatic male Fabry patients and levels were reduced upon therapy. Little is known still about the location of lyso-GSL in plasma. Lyso-GB3 was not detected in lipoproteins, but rather associated with albumin [75]. Lyso-GB3 furthermore inhibited α-galactosidase A activity worsening GB3 storage. It was also shown that lyso-GB3 induced smooth muscle cell proliferation and this extended on studies discussing an unidentified substance in plasma stimulating vascular smooth muscle cells and cardiomyocytes proliferation [75,76]. Studies using human podocytes suggested that lyso-GB3 contributes to Fabry nephropathy [77]. Lyso-GB3 induced TGFβ, invariant chain (CD74) and extracellular matrix (fibronectin and type IV collagen), all components involved in the fibrotic response encountered during kidney derailment [77]. More recently, lyso-GB3, at concentrations occurring in plasma of Fabry patients, was shown to inhibit NO synthase, and thus potentially can contribute to the vasculopathy of the patients [78,79]. Interestingly, activation of the vitamin D receptor, using paricalcitol or calcitriol, counteracted these effects. A later study with human podocytes demonstrated that lyso-GB3 not only provoked a fibrotic response, but also induced inflammation. Monocyte chemoattractant protein-1 (MCP-1/CCL2) and regulated upon activation, normal T cell expressed and presumably secreted (RANTES/CCL5) were induced in human podocytes, which depended on the Notch1 signaling pathway and subsequent activation of NF-κB and could be inhibited by the γ-secretase inhibitor GSI IX [80]. Extracellular matrix formation also required NF-κB activation. Furthermore, PBMCs, monocytes and DC from Fabry patients show increased pro-inflammatory cytokines. It has been suggested that GB3 is a TLR4 recognized ligand, which was experimentally demonstrated in monocyte-derived dendritic cells and macrophages when GB3 was added and simultaneously α-Gal inhibited using 1-deoxygalactonojirimycin (DGJ). This way clearance of GB3 was prevented and increased production of IL-1β and TNFα was observed. Subsequent blocking of TLR4 using antibodies blunted the observed GB3 mediated cytokine induction [81]. If (lyso)-GB3 is also modulating immunity via type II, NKT cells is not yet established [82]. A recent study shows that lyso-GB3 has an impact on growth capacity of bacteria in biofilm assays and in human colon microbiota suspensions. Especially, *Bacteroides fragilis* shows increased outgrowth in individual and multispecies biofilm assays and increased outgrowth in microbiota suspensions. Furthermore, lyso-GB3 modifies the amount of the short-chain fatty acids produced by microbiota, especially butyrate. It is speculated that the striking reduction of butyrate releases the break on histone deacetylase inhibition, allowing for increased inflammation. Concomitantly this may trigger induction of GB3 synthase, thus more lyso-GB3 formation, and may worsen kidney disease and heart disease [83].

## Lyso-gangliosides and lyso-sulfatide

**Figure 4:**
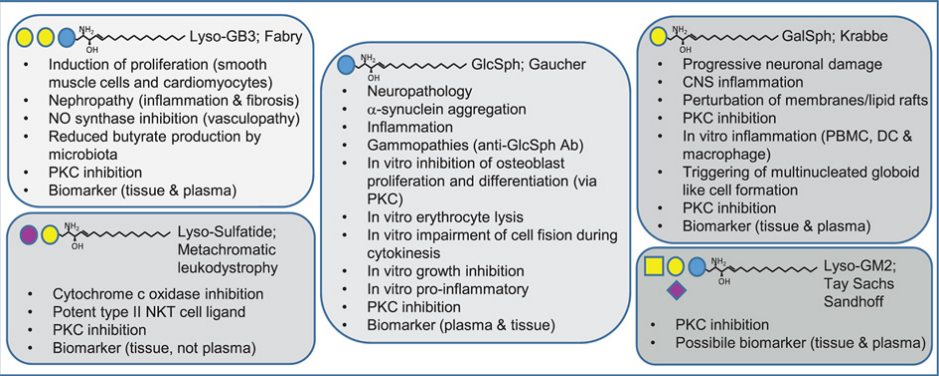
Summary of the actions of the various lyso-GSL species Abbreviations: CNS, central nervous system; DC, dendritic cell; NKT, natural killer T; NO, nitric oxide; PBMC, peripheral blood mononuclear cell; PKC, protein kinase C.

More complex lyso-GSL species are lyso-sulfatides and lyso-gangliosides (lyso-GM1, lyso-GM2, lyso-GA1 and lyso-GA2). Metachromatic leukodystrophy (MLD) is caused by defective lysosomal arylsulfatase A (ASA). Patients and *ASA* gene knockout mice show increased levels of sulfatide and lyso-sulfatide and progressive accumulation of these lipids within oligodendrocytes and Schwann cells is associated with demyelination and axonal loss in the central and peripheral nervous systems [84–86]. Lyso-sulfatide not only inhibits PKC activity, but also has been shown to inhibit cytochrome *c* oxidase and perturb migration of a neuronal precursor cell line [34,84,87]. Interestingly, sulfatide has been shown to be a very potent type II NKT cell ligand and it has for instance been shown that sulfatide activated type II NKT cells may protect against reperfusion damage in liver and against allergic airway inflammation [88–90]. Importantly, *in vitro* studies revealed that lyso-sulfatide is a more potent CD1d-restricted type II NKT cell ligand [91]. In ulcerative colitis, the lamina propria is populated by lyso-sulfatide reactive type II NKT cells, which contribute to local tissue damage [92]. In contrast with lyso-GB3 and GlcSph, lyso-sulfatide does not serve to be a plasma marker suitable for severity and therapy monitoring of MLD [93]. However, lyso-sulfatide levels in sural nerve and cerebrospinal fluid correlate with severity of neuropathy observed in MLD [94]. GM2 accumulation occurs when lysosomal β-hexosaminidase activity is perturbed. This is observed in Tay Sachs Disease and Sandhoff Disease, examples of GM2 gangliosidosis. Recently, lyso-GM2 levels were found to be elevated in plasma of rodent disease models of both diseases. Interestingly, intracerebroventricular injection of modified Hexosaminidase B in Sandhoff disease mouse lowered lyso-GM2 in plasma. Also elevated lyso-GM2 levels were detected in plasma of Tay Sachs and Sandhoff patients and possibly lyso-GM2 may function as biomarker [95]. Additional studies are needed to understand the role of lyso-GM species in LSD in more detail. Figure 4 summarizes the effects of the discussed lyso-GSLs.

## Lyso-GSL good, bad or ugly?

The formation of the deacylated lyso-GSL variants changes the biophysical nature of these lipids allowing lysosomal exit. As a consequence, lysosomal storage load of acylated species will be reduced and potential deleterious lysosome rupture may be avoided. In addition, cellular escape of lyso-species may allow for secretion via urine and bile. It has not been firmly established whether the same holds for other lyso-lipids. A downside of the changed properties is that lyso-species may travel to distant sites causing additional pathology. Possible consequences may be PKC activation, disturbance of membrane micro domains, induction of nerve pain or immune activation. Moreover, when lyso-species chronically trigger CD1d-restricted type II NKT cell activation, this allows for B cell and plasma cell activation and genetic instability due to continuous production of auto-antibodies. In the end, this will lead to gammopathy and malignancy. It is therefore important to break the vicious cycle of ongoing lyso-GSL production in pathology.

## Breaking the vicious lyso-GSL cycle

Given the presumed toxicity of lyso-GSL, it is of interest to consider approaches lowering these compounds. One way for this is to reduce their source, the accumulating corresponding GSLs in lysosomes of cells of LSD patients. Besides enzyme replacement therapies (ERT), pharmacological chaperone therapy (PCT) and envisioned future gene therapies correcting the defective enzyme, substrate reduction therapy (SRT) may be a way to accomplish this [96]. Registered are different inhibitors of GlcCer synthase, the enzyme responsible for formation of GlcCer from which subsequent more complex GSLs are formed [97]. Indeed, SRT has been found to cause reductions in plasma GlcSph in Gaucher patients [98]. In twitcher mice SRT in the form of L-cycloserine, an inhibitor of 3‐ketodyhydrosphingosine synthase, had been shown to lower psychosine and GalCer levels and this treatment prolonged lifespan and delayed pathology [99]. GlcSph lowering has been observed in GD mouse models upon SRT, PCT and lentiviral gene therapy [100–102]. In patients ERT and PCT also lowered GlcSph levels [98,103]. Importantly, GlcSph reduction therapy (SRT with eliglustat) also improved immunological aspects. In GD mice development of B-cell lymphoma and myeloma could be prevented and in another study anti-GlcSph antibodies and clonal immunoglobulin were reduced [65,101]. In GD patients with monoclonal gammopathy of unknown significance reduction in clonal immunoglobulin was observed [68]. In Fabry patients ERT resulted in lowering, but not normalizing, of lyso-GB3 [75]. Alternatively, inhibition of acid ceramidase might be considered. Carmofur, a known inhibitor of the enzyme, is able to reduce the formation of GlcSph in GBA-deficient cells and mice, lysoGB3 in GLA-deficient cells and psychosine in Krabbe patient fibroblast and in Twitcher mice [23–25,48,104,105]. Carmofur is also used as anti-cancer agent and two modes of anti-tumor action have been proposed. AC inhibition, which allows ceramide elevation and connected cell death and generation of 5-FU, a pyrimidine analogue, which causes inhibition of DNA synthesis [23]. Fabrias and colleagues have recently designed more specific acid ceramidase inhibitors [106]. The report that mutations in the *ASAH1* gene impair spinal-cord motor neurons and other areas of the central nervous system suggests that inhibition of acid ceramidase may cause unacceptable side-effects [107]. An alternative approach that showed efficacy in a mouse model of GD was the use of a C5aR antagonist A8 (Δ71−73) to break the vicious C5a-C5aR1 loop held responsible for *UGCG* and GlcCer induction [64].

In conclusion, acid ceramidase activity is essential to generate deacylated lyso-GSL species in the lysosome. By lowering the lysosomal GSL load (ERT, SRT, PCT, or gene therapy), or by inhibiting acid ceramidase activity lyso-GSL pathology could be counteracted.

## Summary

- The importance of lyso-GSLs is increasingly acknowledged as they cause pathology in glycosphingolipidosis.
- In LSDs such as Gaucher Disease, Fabry Disease and Krabbe Disease, lysosomal GSL accumulation occurs. Deacylation of GSL by acid ceramidase results in the formation of lyso-GSLs. Lyso-GSLs can perturb membranes, trigger immune activation, cause severe neuropathology and death due to cancer.
- Understanding exactly how lyso-GSLs contribute to pathology in LSD is key for the development of future therapies. Various GSL lowering therapies exist and GlcSph reduction therapy in GD patients already demonstrated that clonal immunoglobulin lowering occurs. A promising novel alternative could be inhibition of acid ceramidase.

## Abbreviations

α-GalCer: α-galactosylceramide
AICAR: 5-aminoimidazole-4-carboxamide-1-β-D-ribofuranoside
AMPK: AMP-activated protein kinase
APC: antigen presenting cells
ASA: arylsulfatase A
CCL-18: chemokine (C-C motif) ligand 18
CDase: ceramidase
CERS: ceramide synthases
CK: ceramide kinase
CNS: central nervous system
DC: dendritic cell
DES: dihydroceramide desaturase
DGJ: 1-deoxygalactonojirimycin
ER: endoplasmic reticulum
ERT: enzyme replacement therapies
GALC: β-galactosidase
GalCer: galactosylceramide
GalSph: galactosylsphingosine
GD: Gaucher Disease
GlcCer: glucosylceramide
GlcSph: glucosylsphingosine
GPNMB: glycoprotein nonmetastatic melanoma protein B
GSL: glycosphingolipid
HSAN-1: hereditary sensory and autonomic neuropathy type I
IC: immune complexes
KDR: ketosphinganine reductase
Lyso-GSL: lyso-glycosphingolipid
LSDs: lysosomal storage disorders
LPS: lipopolysaccharide
MCP-1: monocyte chemoattractant protein-1
MLD: Metachromatic leukodystrophy
NKT: natural killer T
NO: nitric oxide
NPC: Niemann Pick type C
PBMC: peripheral blood mononuclear cell
PCT: pharmacological chaperone therapy
PKC: protein kinase C
RANTES: regulated upon activation, normal T cell expressed and presumably secreted
SM: sphingomyelin
SO: sphingosine
SPT: serine palmitoyltransferase
SRT: substrate reduction therapy
TCR: T-cell receptor
TLR: toll-like receptor
